# Neuroplasticity and recovery of the brain affected by substance use disorder: multilevel mechanisms and new therapeutic strategies (2020–2025)

**DOI:** 10.3389/fnmol.2026.1760387

**Published:** 2026-02-26

**Authors:** Roberto Estrada-Medina, Berle Estalin Briones-Llamoctanta, Josué Edison Turpo-Chaparro

**Affiliations:** 1 Escuela de Posgrado, Universidad Peruana Unión, Lima, Perú; 2 Escuela Profesional de Psicología, Universidad Peruana Unión, Lima, Perú

**Keywords:** BDNF/TrkB, epigenetics, neuromodulation, neuroplasticity, nucleus accumbens, prefrontal cortex, psychoplastogens, substance use disorder

## Abstract

**Introduction:**

Substance use disorder (SUD) is a complex neurobiological disorder characterized by the consolidation of maladaptive neuroplasticity affecting dopaminergic, glutamatergic, and neurotrophic systems, as well as cortical and subcortical networks critical for executive control, emotional regulation, and associative learning.

**Methods:**

This systematic review was conducted in accordance with PRISMA 2020 guidelines and integrated 57 studies published between 2020 and 2025 to analyze neuroplastic mechanisms involved in vulnerability to substance use disorder and brain recovery following chronic substance exposure.

**Results:**

The findings revealed consistent alterations in synaptic density, BDNF/TrkB signaling, glutamatergic homeostasis, and epigenetic regulation, along with structural and functional neuroimaging changes in regions such as the prefrontal cortex (PFC), nucleus accumbens (NAc), and amygdala. Four core therapeutic domains for neuroplastic restoration were identified: neuromodulation approaches (including repetitive transcranial magnetic stimulation, transcranial direct current stimulation, and deep brain stimulation), compounds that promote neuroplasticity via neurotrophic signaling, epigenetic and anti-inflammatory interventions, and psychological therapies based on memory reconsolidation processes. These strategies demonstrated the capacity to normalize prefrontal activity, modulate reward networks, strengthen emotional regulation, and reduce craving.

**Conclusion:**

Despite significant advances, important gaps remain, including methodological heterogeneity, scarcity of longitudinal studies, and limited clinical generalizability. Overall, the evidence suggests that recovery from substance use disorder requires multimodal interventions simultaneously targeting molecular, synaptic, and circuit-level plasticity, with growing emphasis on personalized approaches guided by neurobiological biomarkers.

## 1 Introduction

Substance use disorders (SUDs) remain among the most persistent global public health challenges, in part because chronic exposure to psychoactive drugs induces long-lasting neurobiological alterations that compromise cognition, emotional regulation, and behavior. A substantial body of contemporary research demonstrates that substance use disorder is fundamentally a disorder of maladaptive neuroplasticity, in which repeated drug exposure reshapes synaptic architecture, reorganizes large-scale neural networks, and biases learning mechanisms toward drug-related cues at the expense of adaptive decision-making (Falade et al., 2025; Raj and Verdejo-García, 2020). These drug-induced alterations manifest across multiple hierarchical levels of brain organization, including epigenetic regulation (He et al., 2020; Iamjan et al., 2021; Wani et al., 2024; Zhang et al., 2022), synaptic potentiation and pruning (Lu et al., 2021; Moliner et al., 2023), corticostriatal connectivity (Le et al., 2021; McConnell et al., 2020), and neuromodulatory balance of reward, salience, and executive control circuits (Ornell et al., 2023; Zhumakhanova et al., 2024; Zolali et al., 2025). Taken together, these findings establish that the brain affected by SUD is not merely damaged, but is actively and systematically reorganized through powerful experience-dependent plasticity.

Dysregulation of the prefrontal cortex (PFC), amygdala, hippocampus, and nucleus accumbens (NAc) is particularly crucial in the transition from controlled substance use to compulsive drug seeking. Neuroimaging studies consistently suggest reduced prefrontal control, impaired inhibitory processing, and pathological strengthening of drug-associated memories—factors that predispose individuals to craving and relapse even after prolonged abstinence (Cole et al., 2020; Le et al., 2021). Molecular evidence complements this perspective by identifying persistent alterations in neurotransmission, particularly glutamatergic remodeling, dopaminergic sensitization, and disrupted signaling of brain-derived neurotrophic factor (BDNF), a key regulator of synaptic plasticity and neuronal survival, which together consolidate addictive behaviors and stabilize them over time (Cole et al., 2020; Memos et al., 2023; Ornell et al., 2023). These neuroadaptations are further reinforced by epigenetic modifications involving transcriptional regulators such as cyclic AMP response element-binding protein (CREB), ΔFosB, methyl-CpG-binding protein 2 (MeCP2), and nuclear factor kappa B (NF-κB), which act as molecular switches encoding long-term vulnerability and shaping both memory systems and affective reactivity associated with drug use (Iamjan et al., 2021; Wani et al., 2024; Zhang et al., 2022).

Importantly, the same neuroplastic mechanisms that underpin SUD also provide a biological substrate for recovery. Accumulating evidence indicates that experiential, pharmacological, and neuromodulatory interventions can reverse or compensate for drug-induced neuroadaptations. Among pharmacological approaches, classic serotonergic psychedelics such as lysergic acid diethylamide (LSD) and psilocin—the active metabolite of psilocybin derived from Psilocybe mushrooms—have been shown to induce rapid and sustained structural and functional plasticity. These compounds act primarily as agonists at serotonin 5-HT2A receptors and, more recently, have been identified as psychoplastogens, a class of compounds capable of promoting synaptic remodeling and network reorganization. Mechanistically, both hallucinogenic and non-hallucinogenic psychoplastogens bind directly to the tropomyosin receptor kinase B (TrkB), thereby enhancing brain-derived neurotrophic factor (BDNF) signaling, which is essential for dendritic growth, synaptic stabilization, and experience-dependent plasticity (Moliner et al., 2023). Consistent with this framework, synthetic non-hallucinogenic analogs such as tabernanthalog (TBG) restore dendritic architecture and normalize stress-related cortical dysfunctions that overlap with vulnerability to SUD (Lu et al., 2021). In parallel, advances in neuromodulation—including repetitive transcranial magnetic stimulation (rTMS), transcranial direct current stimulation (tDCS), and deep brain stimulation (DBS)—demonstrate the capacity to recalibrate frontostriatal circuits, reduce cue reactivity, and improve executive control (Edemann-Callesen et al., 2020; Evancho et al., 2023; Guercio et al., 2020; Kallupi et al., 2022; Liu and Yuan, 2021; Wang et al., 2025). These interventions modulate synaptic strength, alter glutamatergic dynamics, and influence the transcriptomic landscape of key regions such as the nucleus accumbens (NAc), where DBS has been shown to modify the expression of genes involved in synaptic adhesion and intracellular signaling (Cai et al., 2025).

Beyond somatic interventions, psychotherapeutic approaches also harness neuroplasticity by targeting memory reconsolidation, attentional regulation, and the integration of emotional and cognitive processes. Interventions such as mindfulness-based relapse prevention, cognitive behavioral therapy, and eye movement desensitization and reprocessing (EMDR) have been shown to attenuate craving by reshaping neural responses to drug-related cues and modulating functional coupling between the amygdala and the prefrontal cortex (Lomas, 2024; Martínez-Fernández et al., 2024; Romanoff, 2022). In parallel, advances in molecular imaging—particularly positron emission tomography (PET) studies using synaptic and receptor-specific radioligands—enable *in vivo* quantification of synaptic density and receptor-level plasticity, providing objective biomarkers to monitor trajectories of neuroplastic recovery during abstinence and treatment (Ferreira et al., 2025).

Despite these advances, the field remains conceptually fragmented. Much of the existing literature focuses on isolated mechanisms—such as glutamatergic dysregulation, altered brain-derived neurotrophic factor (BDNF) signaling, epigenetic modifications, or circuit-level disconnection—while comparatively few integrative frameworks explain how these processes interact across hierarchical levels to facilitate or constrain recovery. In addition, sex-related differences in neuroplastic responses (Memos et al., 2023; Quigley et al., 2021), the contribution of neuroinflammatory processes to chronic vulnerability (Namba et al., 2021), emerging evidence suggesting that antioxidant and anti-inflammatory compounds may indirectly support neuroplastic recovery through modulation of neuroimmune and neurotrophic signaling, and the role of computational learning mechanisms in relapse dynamics (Gueguen et al., 2021) remain insufficiently characterized. Consequently, a comprehensive synthesis is needed to integrate molecular, cellular, systems-level, and therapeutic perspectives into a coherent model capable of guiding future research and clinical innovation.

This paper addresses this gap by systematically reviewing the evidence from 2020–2025 on the neuroplasticity mechanisms underlying both the deterioration and potential recovery of the SUD-affected brain. Specifically, this review examines molecular pathways, synaptic modifications, circuit-level reorganizations, and neuromodulatory, pharmacological, and behavioral interventions capable of restoring adaptive neuronal function. By integrating findings from animal models, human neuroimaging, epigenetic research, and interventional studies, this synthesis seeks to elucidate the multilayered architecture of addiction-related neuroplasticity and identify convergent mechanisms that could serve as targets for next-generation treatments. Ultimately, understanding how the brain reorganizes itself during SUD and how it can be guided toward recovery remains critical for developing effective, personalized, and mechanistic therapeutic strategies.

## 2 Methodology

This study was conducted in accordance with the methodological guidelines established by the PRISMA 2020 statement for systematic reviews, in order to ensure transparency, completeness, and reproducibility across the procedures of evidence searching, screening, extraction, and synthesis. The methodological approach was designed to identify studies published between January 2020 and March 2025 that addressed neuroplasticity mechanisms linked to SUD and brain recovery processes following exposure to psychoactive substances.

### 2.1 Search strategy

A systematic literature search was performed in four electronic databases: Scopus, Web of Science Core Collection, PubMed/MEDLINE, and ScienceDirect, complemented by manual screening of reference lists from key articles. The search strategy was guided by predefined inclusion and exclusion criteria, as described below. Search terms were selected to capture evidence related to molecular, synaptic, structural, and functional neuroplasticity in the context of SUD. Boolean combinations of keywords were applied, including “neuroplasticity”, “addiction”, “synaptic plasticity”, “substance use disorder”, “neuromodulation”, “brain-derived neurotrophic factor”, “BDNF”, “glutamate”, “epigenetics”, “psychoplastogen”, “deep brain stimulation”, and “relapse vulnerability”. Search strings were adapted to the syntax of each database to maximize sensitivity while preserving specificity.

The systematic search across the four databases yielded 2,347 records. After duplicate removal, 1,962 unique studies remained. These records were screened through title and abstract review, resulting in 127 articles retrieved for full-text assessment based on the predefined criteria, of which 57 studies were ultimately included in the qualitative synthesis.

### 2.2 Inclusion and exclusion criteria

Study eligibility was evaluated using a PICO framework, operationalized for this review as follows: the population comprised human participants or animal models exposed to psychoactive substances; interventions or exposures included neuroplastic mechanisms associated with SUD, withdrawal, or recovery; comparisons involved exposed vs. control groups or contrasts between distinct interventions; and outcomes focused on neuroplastic changes related to vulnerability to substance use disorder (SUD) and recovery.

Studies were included if they met the following criteria: (1) explicitly addressed neuroplasticity in the context of substance use disorder or withdrawal; (2) reported molecular, synaptic, circuit-level, or structural mechanisms associated with brain adaptation or recovery; (3) described relevant interventions, such as neuromodulation, psychoplastogens, epigenetic modulators, memory-based psychotherapy, or biomarkers of plasticity; (4) were published in indexed, peer-reviewed journals; and (5) fell within the established publication period.

Studies were excluded if they (1) did not directly address substance use disorder or neuroplasticity; (2) examined neurological or psychiatric conditions without a clear link to SUD; (3) focused exclusively on ethical, sociotechnical, or epidemiological aspects without neurobiological analysis; or (4) lacked empirical evidence relevant to the objectives of the review. Broad narrative reviews not centered on plasticity mechanisms applicable to addiction were also excluded, except when they provided essential conceptual background for the introduction.

### 2.3 Selection process

**Figure 1:**
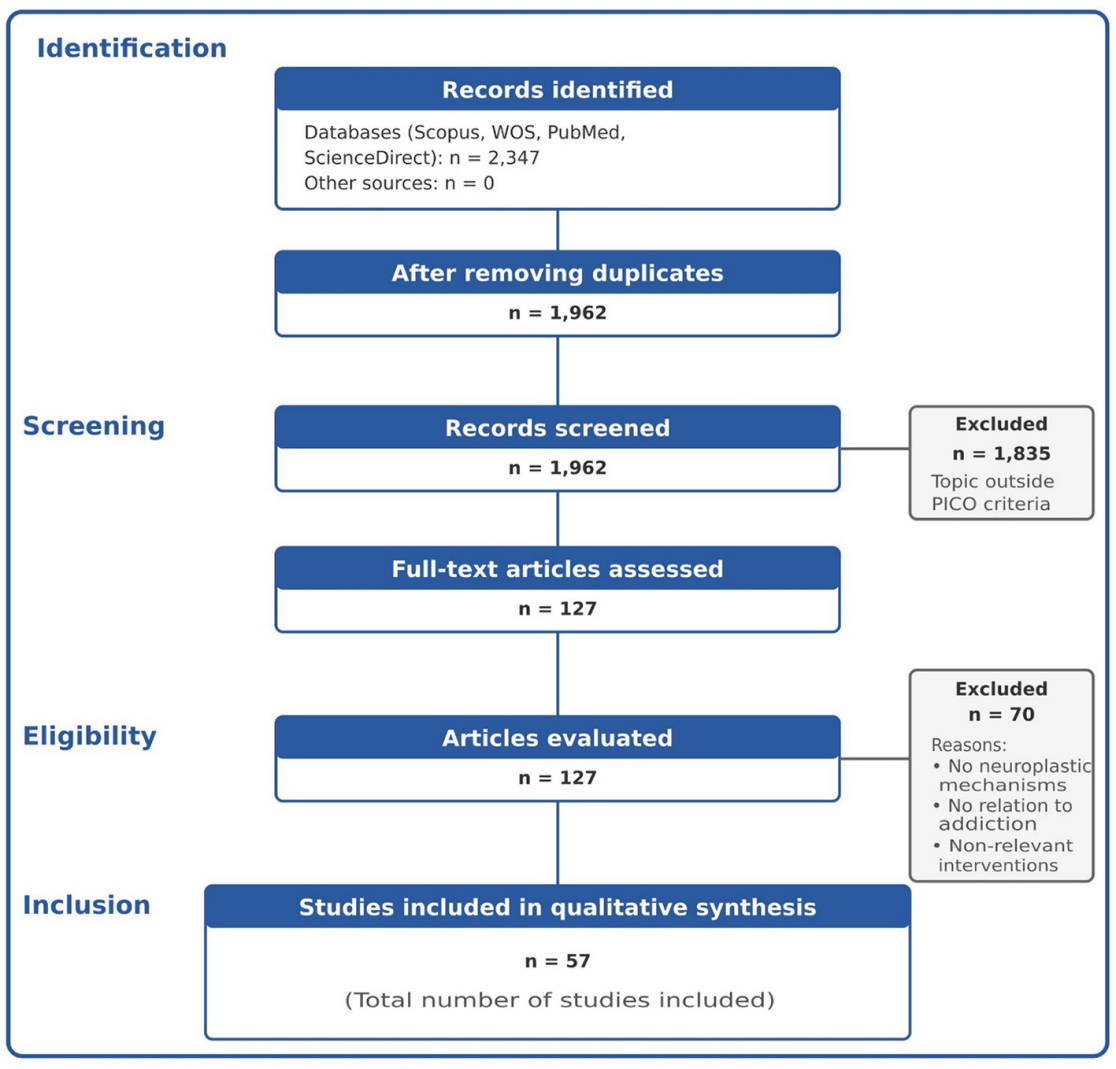
PRISMA diagram.

The 2,347 records initially identified across the four databases underwent a two-phase screening process. In the first phase, after duplicate removal, 1,962 titles and abstracts were assessed for thematic relevance, leading to the exclusion of studies that did not meet the basic eligibility criteria defined by the PICO framework. In the second phase, 127 full-text articles were examined in depth to verify strict compliance with the inclusion criteria related to neuroplasticity mechanisms involved in addiction and recovery. Following this procedure, 57 studies were included in the final qualitative synthesis, corresponding to 30 studies from the first thematic block and 27 from the second, while the remaining articles were excluded for the previously specified methodological and thematic reasons (Figure 1).

### 2.4 Data extraction and synthesis

Data from the included studies were extracted using a standardized matrix designed to capture key information on study type, experimental model, characteristics of the intervention or exposure, brain regions or neurobiological systems involved, neuroplastic mechanisms described, associated biomarkers, and principal findings. This extraction framework enabled organization of the evidence into coherent analytical domains, including molecular, synaptic, epigenetic, circuit-level, and neuroimmune plasticity, as well as modulatory interventions such as psychoplastogens, neuromodulation techniques, memory-based therapies, and antioxidant approaches.

An integrative qualitative synthesis was conducted to identify convergent patterns of neuroplastic alteration and recovery, as well as relevant differences across substances, intervention modalities, experimental models, and stages of the addictive process. In accordance with Frontiers guidelines, a meta-analysis was not performed due to substantial methodological heterogeneity across studies; instead, a rigorous and conceptually articulated narrative integration was prioritized.

### 2.5 Evaluation of methodological quality

Although studies were not excluded solely on the basis of quality, key indicators of methodological rigor—including experimental design, sample size, validity of neuroplasticity measures and biomarkers, and clarity in reporting interventions—were considered to interpret and weight the evidence within the synthesis. Particular emphasis was placed on controlled experimental studies, clinical trials, investigations using advanced neuroimaging or transcriptomic approaches, and previously published systematic reviews that contributed to contextual and methodological interpretation.

### 2.6 Ethical considerations

As this review did not involve primary data collection or direct interaction with human or animal participants, approval from an institutional ethics committee was not required. All sources included in the review are publicly available or accessible through institutional academic subscriptions.

## 3 Results

### 3.1 Resource identification initiative

The analysis of the 57 included studies provides a highly convergent overview of the neuroplasticity mechanisms underlying both vulnerability to SUD and the potential for functional recovery in the SUD-affected brain. Across studies, psychoactive substances were found to induce robust alterations in glutamatergic, dopaminergic, and neurotrophic systems, impacting key regions such as the dorsolateral prefrontal cortex, anterior cingulate cortex, amygdala, and nucleus accumbens. Evidence consistently shows that these changes occur at the synaptic level—through variations in spine density, AMPA receptor redistribution, synaptic potentiation or depression, and altered cortical excitability—and at the molecular level via epigenetic regulation of plasticity-related genes, including BDNF, CREB, ΔFosB, and MeCP2 (He et al., 2020; Iamjan et al., 2021; Memos et al., 2023; Wani et al., 2024; Zhang et al., 2022; Table 1).

**Table 1:** Main findings on neuroplasticity and recovery in addiction (2020–2025).

**Study**	**Mechanism or intervention**	**Region/circuit involved**	**Main finding**	**Relevance to the recovery of the addicted brain**
Memos et al. (2023)	Glutamatergic alterations and cognitive impairment due to methamphetamine	mPFC, striated	Sex-dependent AMPA and GSK3β changes	Explain differential vulnerability and specific targets for executive restoration
Le et al. (2021)	Hypofrontality in consumers	dACC, DLPFC	Meta-analysis confirms reversible executive dysfunction	It reinforces that cortical recovery is possible with targeted interventions
Ceceli et al. (2023)	Volumetric changes in heroin and cocaine addicts	PFC, ventral striatum	Structural reduction associated with inhibitory control	It establishes biomarkers of structural damage and potential recovery
Cole et al. (2020)	BDNF and glutamate alteration in withdrawal	mPFC–striated	Disruption of neurotrophic plasticity	BDNF appears as a central axis in synaptic restoration.
Carboni et al. (2021)	Changes in mRNA during abstinence (tobacco/vaping)	NAc, mPFC	CRF, BDNF dysfunction	Explains the persistence of craving and the need for pro-plasticity interventions
Moliner et al. (2023)	Psychoplastogens (LSD/psilocin) → TrkB activation	Cortex, hippocampus	Rapid and sustained synaptic potentiation	It paves the way for rapid plasticity treatments without adverse effects
Peters and Olson (2021)	Non-hallucinogenic TBG psychoplastogen	Somatosensory cortex	Spine regeneration + normalization of activity	High clinical applicability for restoring damaged circuits
Liu and Yuan (2021)	rTMS in addiction	DLPFC	Improves inhibitory control and reduces craving	Demonstrates the effectiveness of neuromodulation in restoring executive circuits
Eliason et al. (2024)	Neuromodulation in cortical damage	Frontal cortex	Restoration of excitability	It establishes a basis for its use in addiction through similar principles
Guercio et al. (2020)	Infralimbic DBS → cocaine	Infralimbic cortex	Decreases hyperreactive glutamate	Reduces cue-induced relapse; modulates addictive memory
Zhang et al. (2021)	DBS black reticulated substance → methamphetamine	SNr, striatal circuit	Facilitates the extinction of CPP	It weakens drug-reward associations, key to relapse prevention
Kallupi et al. (2022)	DBS accumbens nucleus	NAc, tonsil	Improved mood without reduced consumption	It shows limitations and the need for precise parameterization
Cai et al. (2025)	Post-DBS transcriptomics	NAc	Changes in Nlgn1, Snca, Pde10a	Identifies specific molecular recovery pathways
Zhou et al. (2024)	Glutamatergic alterations in behavioral addiction	dACC, PrL	Elevated glucose associated with compulsiveness	Transdiagnostic mechanism applicable to SUD
Romanoff (2022)	Mindfulness	dACC, attentional networks	Emotional regulation and craving reduction	It complements biological interventions with functional reorganization
Martínez-Fernández et al. (2024)	EMDR in addiction	Amygdala-hippocampus	Memory processing → craving reduction (SMD = −0.866)	Strong evidence of psychological intervention with neuroplastic impact
Wani et al. (2024)	Epigenetic mechanisms (CREB, ΔFosB, MeCP2)	Striated, PFC	They control vulnerability and compulsiveness	It confirms the need for interventions focused on epigenetics
Hammad et al. (2024)	Green tea nanoparticles	Hippocampus	↑BDNF, ↓inflammation	It demonstrates the role of anti-inflammatory drugs in synaptic restoration

The results further indicate that exposure to cocaine, nicotine, opioids, and methamphetamine elicits characteristic patterns of structural and functional reorganization. Structural neuroimaging studies reported reductions in cortical and subcortical volumes associated with impairments in inhibitory control and affective processing (Ceceli et al., 2023; McConnell et al., 2020), whereas functional neuroimaging consistently revealed hypofrontality and dysregulation within salience and reward networks during active consumption and early withdrawal (Le et al., 2021). These alterations were also accompanied by changes in associative learning and memory, particularly within amygdala–striatum circuits, which amplify reactivity to drug-related cues and interfere with extinction-related processes (Guo et al., 2024; Zhang et al., 2021).

Regarding biomarkers, a recurring pattern of dysregulation was observed in BDNF levels, especially during withdrawal from nicotine and other substances, where reduced neurotrophic availability was accompanied by disturbances in glutamatergic signaling within the medial prefrontal cortex and striatum (Carboni et al., 2021; Cole et al., 2020). In addition, transcriptomic evidence suggests that addiction-related molecular adaptations disrupt synaptic homeostasis in a sustained—and potentially transgenerational—manner, supporting the concept of consolidated maladaptive plasticity (Cai et al., 2025; Wani et al., 2024). Importantly, the emergence of *in vivo* approaches for quantifying plasticity, including synaptic density PET imaging, enabled more precise observation of the structural and functional impact of these neurobiological alterations (Ferreira et al., 2025).

With respect to interventions supporting neuroplastic recovery, four major evidence domains were identified: neuromodulation, psychoplastogens, epigenetic approaches, and memory-based psychotherapy. Non-invasive neuromodulation demonstrated relevant effects on executive control and craving reduction. Repetitive transcranial magnetic stimulation targeting the dorsolateral prefrontal cortex restored cortical excitability profiles and improved performance in self-regulation tasks across multiple SUDs (Eliason et al., 2024; Liu and Yuan, 2021). Comparable findings were reported for tDCS, although results were less consistent. In parallel, deep brain stimulation targeting the nucleus accumbens or infralimbic regions modulated circuit activity linked to pathological motivation, facilitated extinction of drug-associated memories, and induced transcriptomic changes suggestive of molecular pathways involved in plasticity restoration (Cai et al., 2025; Guercio et al., 2020; Hassan et al., 2021; Mahoney et al., 2021; Zhang et al., 2021).

A second cluster of evidence derives from psychoplastogens, including LSD, psilocin, and tabernanthalog (TBG), which have shown a notable capacity to enhance TrkB-dependent signaling and promote cortical spinogenesis while avoiding hallucinogenic effects (Aarrestad et al., 2025; Lu et al., 2021; Moliner et al., 2023; Peters and Olson, 2021). Across experimental models, these compounds contributed to the restoration of dendritic architecture and adaptive neural capacity, particularly under chronic stress conditions. These findings suggest that psychoplastogens may reverse components of maladaptive plasticity induced by substance exposure, especially within disrupted prefrontal circuits that sustain compulsive behavioral patterns.

Furthermore, studies examining epigenetic mechanisms reported that regulation of transcription factors such as CREB, ΔFosB, NF-κB, and chromatin-associated proteins plays a pivotal role in the transition to compulsive use and the persistence of addiction-related memories. Interventions targeting these molecular regulators reduced compulsive-like behaviors and promoted restoration of molecular homeostasis, particularly in opioid-related models. Additional evidence indicates that antioxidant compounds, including green tea–derived substances, increase BDNF expression and attenuate neuroinflammatory responses, thereby supporting improved synaptic plasticity during withdrawal (Hammad et al., 2024; Wani et al., 2024).

Finally, several studies emphasized the contribution of memory-based psychological therapies, including EMDR and mindfulness-derived interventions, in modulating frontolimbic networks and reducing craving intensity. Reported effects appear to involve memory reconsolidation processes and functional reorganization of circuits implicated in emotional regulation and attentional control, consistent with neuroplastic recovery dynamics beyond somatic interventions (Lomas, 2024; Martínez-Fernández et al., 2024; Romanoff, 2022).

Taken together, the evidence suggests that recovery in the SUD-affected brain is not explained by a single pathway, but rather by convergent processes that restore structural and functional plasticity at multiple levels: molecular, synaptic, circuit, and behavioral. Despite methodological heterogeneity, findings consistently support that normalization of prefrontal function, modulation of glutamatergic transmission, restoration of BDNF/TrkB signaling, and reconfiguration of associative memory networks represent key pillars of sustained neurobiological recovery.

## 4 Discussion

The findings of this systematic review enable a more precise and integrative understanding of the neurobiological mechanisms governing both vulnerability to SUD and the potential for recovery in the SUD-affected brain. In line with contemporary neuroscience models of addiction, the reviewed evidence confirms that repeated substance exposure induces profound alterations in synaptic and molecular plasticity systems, reinforcing maladaptive adaptations that disrupt associative learning, increase reactivity to drug-related cues, impair emotional regulation, and weaken executive control (Guo et al., 2024; Le et al., 2021; Memos et al., 2023; Raj and Verdejo-García, 2020). These processes, reflected in structural changes detected through neuroimaging, glutamatergic and dopaminergic dysregulation, and disturbances in neurotrophic signaling, may explain the persistence of SUD-related compulsive drug seeking and relapse risk even after extended periods of abstinence (Ceceli et al., 2023; Cole et al., 2020; McConnell et al., 2020).

A key emerging contribution is the relevance of BDNF and TrkB signaling as a shared axis linking neuroplastic deterioration and recovery. Multiple studies indicate that withdrawal—particularly from nicotine and cocaine—is associated with reduced BDNF expression and disrupted glutamatergic balance in critical regions such as the medial prefrontal cortex and striatum (Carboni et al., 2021; Cole et al., 2020). This reduction may compromise synaptic recalibration capacity and sustain compulsive behavioral patterns. In this context, evidence on psychoplastogens—including LSD, psilocin, and the non-hallucinogenic compound tabernanthalog—supports the view that sustained TrkB activation can rapidly restore spine density and normalize cortical dynamics even under chronic stress conditions (Aarrestad et al., 2025; Lu et al., 2021; Moliner et al., 2023; Peters and Olson, 2021). Collectively, these findings suggest that interventions enhancing the BDNF/TrkB pathway may represent a promising mechanistic platform for next-generation addiction treatments.

This review also reinforces the central role of the glutamatergic system in both SUD maintenance and recovery. The literature consistently documents disrupted glutamatergic homeostasis across prefrontostriatal and cingulate circuits, which contributes to reduced inhibitory control and the progression toward compulsive substance use characteristic of SUD (Le et al., 2021; Memos et al., 2023; Zhou et al., 2024). Interventions targeting these networks—such as rTMS and transcranial direct current stimulation (tDCS)—demonstrated the capacity to normalize cortical excitability patterns, improve self-regulation, and reduce craving across multiple SUDs (Eliason et al., 2024; Liu and Yuan, 2021). Although variability across studies remains substantial, the overall evidence supports neuromodulation as a relevant component, particularly during treatment phases aimed at strengthening executive function and relapse prevention.

In parallel, deep brain stimulation (DBS) is emerging as a circuit-level intervention capable of modulating subcortical networks involved in pathological motivation. The studies analyzed suggest that stimulation of the nucleus accumbens, infralimbic cortex, or substantia nigra can modify activity within circuits associated with compulsive drug seeking, facilitate extinction of drug-related memory traces, and induce transcriptomic adaptations consistent with plasticity restoration mechanisms (Cai et al., 2025; Guercio et al., 2020; Hassan et al., 2021; Mahoney et al., 2021; Zhang et al., 2021). However, the evidence is not fully uniform: in some experimental settings, DBS reduced emotional withdrawal symptoms but did not decrease substance self-administration (Kallupi et al., 2022). This pattern indicates that DBS efficacy may depend on stimulation parameters, baseline circuit state, stage of SUD, and substance-specific neuroadaptations, warranting more standardized translational evaluation.

Epigenetic research provides an essential framework for interpreting SUD as a form of stable molecular reprogramming mediated by transcriptional regulators such as CREB, ΔFosB, MeCP2, and NF-κB (Wani et al., 2024). These mechanisms may sustain long-term gene-expression shifts that reinforce negative affective states and preserve relapse vulnerability even in the absence of ongoing drug exposure. Importantly, identifying epigenetic mediators of maladaptive plasticity also opens opportunities for targeted interventions aimed at restoring regulatory balance, particularly in opioid-related models where compulsive patterns and relapse risk remain high.

In addition, the literature highlights psychological interventions grounded in memory and awareness processes, including EMDR and mindfulness-based approaches, which demonstrated effects on the reorganization of frontolimbic networks and the attenuation of craving (Lomas, 2024; Martínez-Fernández et al., 2024; Romanoff, 2022). These outcomes appear to involve mechanisms of memory reconsolidation and emotion regulation, supporting the premise that psychological and neurobiological pathways of recovery are closely interdependent. This aligns with integrative models conceptualizing SUD as an emergent disorder shaped by interactions between synaptic plasticity, affective memory consolidation, and executive regulatory systems.

Despite these advances, the evidence synthesized in this review also reveals substantial gaps. First, many neurobiological interventions have been tested primarily in animal models or small clinical samples, limiting external validity and clinical generalizability. Second, heterogeneity across substances, designs, and neuroplasticity measures complicates direct cross-study comparisons and restricts the formulation of unified mechanistic conclusions. Third, longitudinal evidence remains limited, particularly regarding the persistence of neuroplastic recovery markers beyond short follow-up windows. Finally, although emerging research underscores the modulatory role of biological sex in vulnerability and recovery trajectories—especially in methamphetamine-related models—this variable remains inconsistently reported and insufficiently integrated into addiction neuroplasticity research (Memos et al., 2023; Quigley et al., 2021).

Nevertheless, the available evidence converges on the view that recovery of the SUD-affected brain requires interventions capable of normalizing prefrontal activity, re-establishing glutamatergic balance, restoring BDNF/TrkB signaling, and modulating affective memory networks associated with substance-related cues. The integration of multimodal strategies—combining neuromodulation, psychoplastogens, memory-based therapies, and epigenetic or anti-inflammatory interventions—appears to represent the most promising direction for future therapeutic development. This convergence strengthens current mechanistic models of addiction and highlights the need for rigorous clinical trials evaluating combined treatments and identifying neuroplastic recovery biomarkers to guide personalized interventions.

## 5 Conclusion

This systematic review integrated recent evidence to delineate the neurobiological mechanisms underlying recovery in the SUD-affected brain, highlighting that this process depended on the convergence of neuroplastic adaptations across multiple levels. The studies analyzed confirmed that substance use disorder induced profound molecular, synaptic, and circuit reorganization, characterized by disrupted glutamatergic homeostasis, dopaminergic dysregulation, reduced BDNF/TrkB signaling, and sustained epigenetic modifications that perpetuated compulsiveness, negative affect, and vulnerability to relapse. These maladaptive mechanisms compromised the brain's capacity to recalibrate functional connectivity and helped explain the persistence of the disorder even after prolonged periods of abstinence.

At the same time, the literature reviewed demonstrated that meaningful therapeutic windows existed for reversing, compensating for, or modulating maladaptive plasticity. Interventions including non-invasive neuromodulation, deep brain stimulation, psychoplastogens, memory-based psychological therapies, and epigenetic-modulating approaches showed consistent effects on strengthening executive control, restoring glutamatergic balance, and improving prefrontal synaptic integrity. Among these strategies, enhancement of the BDNF/TrkB axis and modulation of frontostriatal circuits emerged as particularly promising mechanistic pathways for functional restoration.

Nevertheless, substantial challenges remained. Methodological heterogeneity, the limited availability of well-powered clinical trials, and the scarcity of longitudinal evidence constrained precise conclusions regarding the durability of neuroplastic recovery. Moreover, key modifiers such as biological sex, substance exposure history, individual vulnerability, and substance-specific neuroadaptations required more systematic investigation to advance toward truly personalized interventions. Despite these limitations, the findings synthesized in this review provided a robust foundation for developing multimodal strategies integrating biological, psychological, and circuit-level mechanisms of recovery.

Taken together, this review underscored that recovery in the SUD-affected brain was not a passive return to pre-use homeostasis, but rather an active and dynamic process of neurobiological reorganization that could be facilitated through targeted, evidence-based interventions. Clarifying the interdependence between molecular, synaptic, and network mechanisms was expected to support the design of more effective, precise, and sustainable therapeutic approaches, contributing to advances in the treatment of SUDs.

## Data Availability

The original contributions presented in the study are included in the article/Supplementary material, further inquiries can be directed to the corresponding author.

## References

[B1] Aarrestad I. K. Cameron L. P. Fenton E. M. Casey A. B. Rijsketic D. R. Patel S. D. . (2025). The psychoplastogen tabernanthalog induces neuroplasticity without proximate immediate early gene activation. Nat. Neurosci. 28, 1919–1931. doi: 10.1038/s41593-025-02021-1 40760185 PMC12373294

[B2] Cai C. Gao L. Zhu Z. Chen W. Zhang F. Yu C. . (2025). Change in brain molecular landscapes following electrical stimulation of the nucleus accumbens. Neuropsychopharmacology. doi: 10.1101/2024.09.30.615737 40999234 PMC12708816

[B3] Carboni L. Ponzoni L. Braida D. Sala M. Gotti C. Zoli M. (2021). Altered mRNA levels of stress-related peptides in mouse hippocampus and caudate–putamen in withdrawal after long-term intermittent exposure to tobacco smoke or electronic cigarette vapour. Int. J. Mol. Sci. 22, 1–17. doi: 10.3390/ijms22020599 33435320 PMC7827390

[B4] Ceceli A. O. Huang Y. Kronberg G. Malaker P. Miller P. King S. G. . (2023). Common and distinct fronto-striatal volumetric changes in heroin and cocaine use disorders. Brain 146, 1662–1671. doi: 10.1093/brain/awac366 36200376 PMC10319776

[B5] Cole R. D. Zimmerman M. Matchanova A. Kutlu M. G. Gould T. J. Parikh V. (2020). Cognitive rigidity and BDNF-mediated frontostriatal glutamate neuroadaptations during spontaneous nicotine withdrawal. Neuropsychopharmacology 45, 866–876. doi: 10.1038/s41386-019-0574-6 31752015 PMC7075915

[B6] Edemann-Callesen H. Barak S. Hadar R. Winter C. (2020). Choosing the optimal brain target for neuromodulation therapies as alcohol addiction progresses—insights from pre-clinical studies. Curr. Addict. Rep. 7, 237–244. doi: 10.1007/s40429-020-00316-w

[B7] Eliason M. Kalbande P. P. Saleem G. T. (2024). Is non-invasive neuromodulation a viable technique to improve neuroplasticity in individuals with acquired brain injury? A review. Front. Hum. Neurosci. 18:1341707. doi: 10.3389/fnhum.2024.1341707 39296918 PMC11408216

[B8] Evancho A. Tyler W. J. McGregor K. (2023). A review of combined neuromodulation and physical therapy interventions for enhanced neurorehabilitation. Front. Hum. Neurosci. 17:1151218. doi: 10.3389/fnhum.2023.1151218 37545593 PMC10400781

[B9] Falade J. Ajiboye O. Olaogun D. Anadu V. Momodu I. Ola O. (2025). Neuroanatomical basis of addiction: a narrative review. East Afr. J. Neurol. Sci. 4, 63–79. doi: 10.4314/eajns.v4i1.8

[B10] Ferreira M. Matias F. Abrunhosa A. Martins R. Castelo-Branco M. (2025). Evidence for neuroplasticity in the human brain in health and disease: a systematic review focusing on molecular imaging using PET. Neural Plast. 2025:9423232. doi: 10.1155/np/9423232

[B11] Gueguen M. C. Schweitzer E. M. Konova A. B. (2021). Computational theory-driven studies of reinforcement learning and decision-making in addiction: what have we learned? Curr. Opin. Behav. Sci. 38, 40–48. doi: 10.1016/j.cobeha.2020.08.007 34423103 PMC8376201

[B12] Guercio L. A. Wimmer M. E. Schmidt H. D. Swinford-Jackson S. E. Pierce R. C. Vassoler F. M. (2020). Deep brain stimulation of the infralimbic cortex attenuates cocaine priming-induced reinstatement of drug seeking. Brain Res. 1746:147011. doi: 10.1016/j.brainres.2020.147011 32652146 PMC7484137

[B13] Guo X. Yuan Y. Su X. Cao Z. Chu C. Lei C. . (2024). Different projection neurons of basolateral amygdala participate in the retrieval of morphine withdrawal memory with diverse molecular pathways. Mol. Psychiatry 29, 793–808. doi: 10.1038/s41380-023-02371-x 38145987 PMC11153146

[B14] Hammad A. M. Alzaghari L. F. Alfaraj M. Lux V. Sunoqrot S. (2024). Green tea polyphenol nanoparticles reduce anxiety caused by tobacco smoking withdrawal in rats by suppressing neuroinflammation. Toxics 12:598. doi: 10.3390/toxics12080598 39195700 PMC11360476

[B15] Hassan O. Phan S. Wiecks N. Joaquin C. Bondarenko V. (2021). Outcomes of deep brain stimulation surgery for substance use disorder: a systematic review. Neurosurg. Rev. 44, 1967–1976. doi: 10.1007/s10143-020-01415-y 33037538

[B16] He L. Liao Y. Wu Q. Liu T. (2020). Association between brain-derived neurotrophic factor Val66Met polymorphism and methamphetamine use disorder: a meta-analysis. Front. Psychiatry 11:585852. doi: 10.3389/fpsyt.2020.585852 33329128 PMC7716815

[B17] Iamjan S. A. Thanoi S. Watiktinkorn P. Fachim H. Dalton C. F. Nudmamud-Thanoi S. . (2021). Changes of exon IV DNA methylation are associated with methamphetamine dependence. Epigenomics 13, 953–965. doi: 10.2217/epi-2020-0463 34008409

[B18] Kallupi M. Kononoff J. Melas P. A. Qvist J. S. de Guglielmo G. Kandel E. R. . (2022). Deep brain stimulation of the nucleus accumbens shell attenuates cocaine withdrawal but increases cocaine self-administration, cocaine-induced locomotor activity, and GluR1/GluA1 in the central nucleus of the amygdala in male cocaine-dependent rats. Brain Stimul. 15, 13–22. doi: 10.1016/j.brs.2021.11.003 34742997 PMC8816878

[B19] Le T. M. Potvin S. Zhornitsky S. Li C. S. R. (2021). Distinct patterns of prefrontal cortical disengagement during inhibitory control in addiction: a meta-analysis based on population characteristics. Neurosci. Biobehav. Rev. 127, 255–269. doi: 10.1016/j.neubiorev.2021.04.028 33933507 PMC8411123

[B20] Liu Q. Yuan T. (2021). Noninvasive brain stimulation of addiction: one target for all? Psychoradiology 1, 172–184. doi: 10.1093/psyrad/kkab016 38666219 PMC10917190

[B21] Lomas C. (2024). Neurobiology, psychotherapeutic interventions, and emerging therapies in addiction: a systematic review. J. Addict. Dis. doi: 10.1080/10550887.2024.2440184 39690473

[B22] Lu J. Tjia M. Mullen B. Cao B. Lukasiewicz K. Shah-Morales S. . (2021). An analog of psychedelics restores functional neural circuits disrupted by unpredictable stress. Mol. Psychiatry 26, 6237–6252. doi: 10.1038/s41380-021-01159-1 34035476 PMC8613316

[B23] Mahoney J. J. Haut M. W. Hodder S. L. Zheng W. Lander L. R. Berry J. H. . (2021). Deep brain stimulation of the nucleus accumbens/ventral capsule for severe and intractable opioid and benzodiazepine use disorder. Exp. Clin. Psychopharmacol. 29, 210–215. doi: 10.1037/pha0000453 34043402 PMC8422285

[B24] Martínez-Fernández D. E. Fernández-Quezada D. Garzón-Partida A. P. Aguilar-García I. G. García-Estrada J. Luquín S. (2024). The effect of eye movement desensitization and reprocessing (EMDR) therapy on reducing craving in populations with substance use disorder: a meta-analysis. Brain Sci. 14:1110. doi: 10.3390/brainsci14111110 39595873 PMC11592247

[B25] McConnell P. A. Garland E. L. Zubieta J. K. Newman-Norlund R. Powers S. Froeliger B. (2020). Impaired frontostriatal functional connectivity among chronic opioid using pain patients is associated with dysregulated affect. Addict. Biol. 25:e12743. doi: 10.1111/adb.12743 30945801 PMC6776713

[B26] Memos N. Avila J. A. Rodriguez E. Serrano P. A. (2023). Synaptic remodeling of GluA1 and GluA2 expression in the nucleus accumbens promotes susceptibility to cognitive deficits concomitant with downstream GSK3β mediated neurotoxicity in female mice during abstinence from voluntary oral methamphetamine. Addict. Neurosci. 8:100112. doi: 10.1016/j.addicn.2023.100112 37842014 PMC10569060

[B27] Moliner R. Girych M. Brunello C. A. Kovaleva V. Biojone J. Enkavi G. . (2023). Psychedelics promote plasticity by directly binding to BDNF receptor TrkB. Nat. Neurosci. 26, 1032–1041. doi: 10.1038/s41593-023-01316-5 37280397 PMC10244169

[B28] Namba M. D. Leyrer-Jackson J. M. Nagy E. K. Olive M. F. Neisewander J. L. (2021). Neuroimmune mechanisms as novel treatment targets for substance use disorders and associated comorbidities. Front. Neurosci. 15:650785. doi: 10.3389/fnins.2021.650785 33935636 PMC8082184

[B29] Ornell F. Scherer J. N. Schuch J. B. Sordi A. O. Halpern S. C. Rebelatto F. P. . (2023). Serum BDNF levels increase during early drug withdrawal in alcohol and crack cocaine addiction. Alcohol 111, 1–7. doi: 10.1016/j.alcohol.2023.04.001 37037287

[B30] Peters J. Olson D. E. (2021). Engineering safer psychedelics for treating addiction. Neurosci. Insights 16:26331055211033847. doi: 10.1177/26331055211033847 34350400 PMC8295933

[B31] Quigley J. A. Logsdon M. K. Turner C. A. Gonzalez I. L. Leonardo N. B. Becker J. B. (2021). Sex differences in vulnerability to addiction. Neuropharmacology 187:108491. doi: 10.1016/j.neuropharm.2021.108491 33567305 PMC7979496

[B32] Raj K. Verdejo-García A. (2020). “From impulses to compulsions,” in Cognition and Addiction: A Researcher's Guide from Mechanisms Towards Interventions (Elsevier: Academic Press), 9–15. doi: 10.1016/B978-0-12-815298-0.00002-2

[B33] Romanoff S. R. (2022). Commentary on mindfulness-based techniques for behavioral addiction. Clin. Psychol. Sci. Pract. 29, 397–399. doi: 10.1037/cps0000109

[B34] Wang K. Li Y. Zhang T. Liu H. Luo J. (2025). Potential benefits and mechanisms of physical exercise and rTMS in improving brain function in people with drug use disorders. Gen. Hosp. Psychiatry 93, 61–66. doi: 10.1016/j.genhosppsych.2025.01.001 39826308

[B35] Wani S. N. Grewal A. K. Khan H. Singh T. G. (2024). Elucidating the molecular symphony: unweaving the transcriptional and epigenetic pathways underlying neuroplasticity in opioid dependence and withdrawal. Psychopharmacology 241, 1955–1981. doi: 10.1007/s00213-024-06684-9 39254835

[B36] Zhang L. Meng S. Chen W. Chen Y. Huang E. Zhang G. . (2021). High-frequency deep brain stimulation of the substantia nigra pars reticulata facilitates extinction and prevents reinstatement of methamphetamine-induced conditioned place preference. Front. Pharmacol. 12:705813. doi: 10.3389/fphar.2021.705813 34276387 PMC8277946

[B37] Zhang R. Dang W. Zhang J. He R. Li G. Zhang L. . (2022). Methylation quantitative locus rs3758653 in the DRD4 gene is associated with duration from first heroin exposure to addiction. Brain Res. 1775:147746. doi: 10.1016/j.brainres.2021.147746 34864042

[B38] Zhou H. Hong T. Chen X. Su C. Teng B. Xi W. . (2024). Glutamate concentration of medial prefrontal cortex is inversely associated with addictive behaviors: a translational study. Transl. Psychiatry. 14:3145. doi: 10.1038/s41398-024-03145-x 39396023 PMC11470925

[B39] Zhumakhanova R. Zhaparkulova N. Sharipova S. Orazbayeva N. Yeszhan B. Zhaksybay Z. . (2024). Innovative achievements in the detection of synaptic plasticity and oxidative stress in mice: precision imaging, improved biosensoring, and personalized interventions for neurological disorders. Casp. J. Environ. Sci. 22, 697–713.

[B40] Zolali E. McMahon L. R. Obeng S. (2025). “Effects of alcohol and opioids on neuroregeneration,” in Tissue Repair and Regeneration: Elucidating Cellular and Molecular Mechanisms with Therapeutic Implications (Cham: Springer Nature Switzerland), 263–280. doi: 10.1007/978-3-031-93677-7_12

