# Supplementary material for: Neuroplasticity and recovery of the brain affected by substance use disorder: multilevel mechanisms and new therapeutic strategies (2020–2025)
Source: Front Mol Neurosci. 2026 Feb 26;19:1760387. doi: 10.3389/fnmol.2026.1760387 (PMC12979374; doi:10.3389/fnmol.2026.1760387)
Supplement: Supplementary file 1 [file Supplementary_file_1.docx]

Supplementary Table S1. Studies included in the systematic review (2020–2025)

| **No.** | **Author(s) & Year** | **Key Mechanism / Domain** | **Main Finding / Neurobiological Relevance** |
| --- | --- | --- | --- |
| 1 | Aarrestad et al. (2025) | Psychoplastogens | TrkB pathway activation and rapid cortical plasticity enhancement. |
| 2 | Cai et al. (2025) | DBS / Transcriptomics | Alterations in synaptic adhesion genes (Nlgn1) within the NAc. |
| 3 | Falade et al. (2025) | Maladaptive Plasticity | Synaptic architecture remodeling induced by chronic drug exposure. |
| 4 | Ferreira et al. (2025) | Synaptic Density (PET) | In vivo quantification of synaptogenesis during recovery phases. |
| 5 | Jain (2025) | Functional Rehab | Role of meaningful activities in promoting neural recovery. |
| 6 | Wang et al. (2025) | Neuromodulation | Recalibration of dysfunctional frontostriatal circuits. |
| 7 | Zolali et al. (2025) | Neuromodulatory Balance | Reconfiguration of reward and executive control networks. |
| 8 | Eliason et al. (2024) | rTMS / Neuromodulation | Restoring excitability and top-down control in the frontal cortex. |
| 9 | Lenze et al. (2021) | Precision Clinical Trials | A new framework for individualized treatment in neurobehavioral and addictive disorders. |
| 10 | Guo et al. (2024) | Associative Memory | Strengthening of cue-reactivity within amygdala-striatal circuits. |
| 11 | Hammad et al. (2024) | Nanoparticles / Antioxidants | BDNF upregulation and reduction of neuroinflammation. |
| 12 | Lomas (2024) | Mindfulness | Modulation of frontolimbic networks and craving reduction. |
| 13 | Martínez-Fernández et al. (2024) | EMDR | Trauma processing and memory reconsolidation with plastic impact. |
| 14 | Stankevich et al. (2024) | Systemic Adaptation | Brain organizational changes as a result of adaptation to insults. |
| 15 | Wani et al. (2024) | Epigenetics | Role of CREB and ΔFosB in the persistence of addictive memories. |
| 16 | Zhou et al. (2024) | Glutamate (MRS) | Medial PFC glutamate levels inversely associated with compulsion. |
| 17 | Zhumakhanova et al. (2024) | Synaptic Biosensors | Detection of oxidative stress impact on synaptic plasticity. |
| 18 | Ceceli et al. (2023) | Structural Neuroimaging | Cortical atrophy linked to impaired impulse control. |
| 19 | Evancho et al. (2023) | Focal Neuromodulation | Executive control enhancement via targeted brain stimulation. |
| 20 | Memos et al. (2023) | Sexual Dimorphism | Sex-dependent AMPA/GSK3β alterations in the mPFC. |
| 21 | Moliner et al. (2023) | TrkB Receptors | Psychoplastogen-induced structural plasticity without hallucinations. |
| 22 | Ornell et al. (2023) | Neurotransmission | Dopaminergic sensitization and trophic signaling in craving. |
| 23 | Romanoff (2022) | Mindfulness | Functional reorganization of attentional and salience networks. |
| 24 | Zhang et al. (2022) | DNA Methylation (DRD4) | Epigenetic markers associated with the speed of addiction onset. |
| 25 | Carboni et al. (2021) | mRNA / Withdrawal | Dysregulation of BDNF and CRF in the NAc during abstinence. |
| 26 | Gueguen et al. (2021) | Computational Models | Reinforcement learning dynamics in addiction and relapse cycles. |
| 27 | Hassan et al. (2021) | Deep Brain Stimulation | Modulation of pathological motivation in limbic circuits. |
| 28 | Iamjan et al. (2021) | Epigenetics (MeCP2) | Transcriptional switches governing chronic vulnerability. |
| 29 | Le et al. (2021) | Hypofrontality | Meta-analysis on reversible executive dysfunction in dACC/DLPFC. |
| 30 | Liu & Yuan (2021) | rTMS (PFC) | Inhibitory control improvement and reduced clinical relapse. |
| 31 | Lu et al. (2021) | Psychoplastogens (TBG) | Regeneration of dendritic spine complexity in cortical neurons. |
| 32 | Mahoney et al. (2021) | Clinical DBS | Therapeutic application of DBS in substance use disorders. |
| 33 | Namba et al. (2021) | Neuroinflammation | Glial cell involvement in maladaptive neuroplasticity. |
| 34 | Peters & Olson (2021) | Psychoplastogens | Restoration of damaged neural circuits via chemical induction. |
| 35 | Quigley et al. (2021) | Biological Sex | Differential neuroplastic responses between males and females. |
| 36 | Zhang et al. (2021) | DBS (Substantia Nigra) | Facilitating extinction of drug-reward associative memories. |
| 37 | Cole et al. (2020) | BDNF / Glutamate | Disruption of trophic plasticity in mPFC-striatal pathways. |
| 38 | Edemann-Callesen et al. (2020) | Neuromodulation (tDCS) | Use of direct current to recalibrate addiction-related circuits. |
| 39 | Guercio et al. (2020) | DBS (Infralimbic) | Reduction of cue-induced relapse through circuit modulation. |
| 40 | He et al. (2020) | Molecular Plasticity | Hierarchical regulation of gene expression in addictive states. |
| 41 | McConnell et al. (2020) | Functional Connectivity | Alterations in corticostriatal communication and habit formation. |
| 42 | Raj & Verdejo-Garcia (2020) | Decision Making | Dysregulation of adaptive learning mechanisms in addiction. |
| 43 | Kallupi et al. (2022) | DBS (NAc) | Impact on negative affect and withdrawal-induced anxiety. |
| 44 | Martinotti et al. (2020) | Neuromodulation (rTMS) | Comprehensive review of rTMS efficacy in reducing craving and relapse. |
| 45 | Mehta et al. (2024) | Neuromodulation Meta-analysis | Systematic evaluation of invasive and non-invasive brain stimulation in SUD. |
| 46 | Takamatsu et al. (2024) | Physical Exercise | Exercise-induced modulation of hippocampal BDNF mRNA expression. |
| 47 | Tataranu & Rizea (2025) | Cellular Mechanisms | Comprehensive review of therapeutic advances in neuroplastic recovery. |
| 48 | Tortora et al. (2024) | Virtual Reality (VR) | Cognitive rehabilitation through immersive environments to enhance plasticity. |
| 49 | Ulisse et al. (2025) | Emerging Interventions | Narrative review on the synergy between psychedelics and neuromodulation. |
| 50 | Yang et al. (2025) | Precision Lesioning | Translating OCD circuit-based solutions to addiction therapeutic targets. |
| 51 | Yin et al. (2021) | Combined Therapy | Synergistic effects of rTMS and exercise on neuronal plasticity. |
| 52 | Acurio Padilla et al. (2024) | Neurological Rehab | Importance of structured rehabilitation in long-term neural adaptation. |
| 53 | Martínez-Fernández et al. (2024) | Optogenetics | Real-time control of drug-seeking circuitry in animal models. |
| 54 | Carlon et al. (2025) | Psychological Interventions | Meta-analysis on positive psychology's role in addiction recovery. |
| 55 | Gazerani (2025) | Neuroplastic Frontiers | Critical review of current breakthroughs in brain adaptation and recovery. |
| 56 | Jahn et al. (2021) | Immersive VR | Evidence of cognitive enhancement in psychiatric disorders via virtual training. |
| 57 | Keady et al. (2024) | Extinction Learning | Sex differences in fear and addiction-related extinction after nicotine use. |

**References**

Aarrestad, I. K., Cameron, L. P., Fenton, E. M., Casey, A. B., Rijsketic, D. R., Patel, S. D., Sambyal, R., Johnson, S. B., Ly, C., Viswanathan, J., Barragan, E. V., Lozano, S. A., Seban, N., Hu, H., Powell, N. A., Chytil, M., Meyer, R., Rose, D., Hempel, C., … Olson, D. E. (2025). The psychoplastogen tabernanthalog induces neuroplasticity without proximate immediate early gene activation. *Nature Neuroscience*, *28*(9), 1919–1931. https://doi.org/10.1038/s41593-025-02021-1

Acurio Padilla, P. E., Latorre Barragán, F., Altamirano Guerrero, O. E., & Barragán Martínez, C. E. (2024). Neural Plasticity after Traumatic Brain Injuries. Importance of neurological rehabilitation[Plasticidad Neural tras Lesiones Traumáticas Cerebrales. Importancia de la rehabilitación neurológica]. *Salud, Ciencia y Tecnologia - Serie de Conferencias*, *3*. https://doi.org/10.56294/SCTCONF2024.771

Cai, C., Gao, L., Zhu, Z., Chen, W., Zhang, F., Yu, C., Xu, K., Zhu, J., & Wu, H. (2025). Change in brain molecular landscapes following electrical stimulation of the nucleus accumbens. *Neuropsychopharmacology*. https://doi.org/10.1038/s41386-025-02241-w

Carboni, L., Ponzoni, L., Braida, D., Sala, M., Gotti, C., & Zoli, M. (2021). Altered mrna levels of stress-related peptides in mouse hippocampus and caudate-putamen in withdrawal after long-term intermittent exposure to tobacco smoke or electronic cigarette vapour. *International Journal of Molecular Sciences*, *22*(2), 1–17. https://doi.org/10.3390/ijms22020599

Carlon, H. A., Hurlocker, M. C., Hoeppner, B. B., & Witkiewitz, K. (2025). Positive psychological interventions for substance use, addiction and recovery: An updated systematic review and meta-analysis. *Addiction*, *120*(7), 1295–1324. https://doi.org/10.1111/add.70019

Ceceli, A. O., Huang, Y., Kronberg, G., Malaker, P., Miller, P., King, S. G., Gaudreault, P. O., Mcclain, N., Gabay, L., Vasa, D., Newcorn, J. H., Ekin, D., Alia-Klein, N., & Goldstein, R. Z. (2023). Common and distinct fronto-striatal volumetric changes in heroin and cocaine use disorders. *Brain*, *146*(4), 1662–1671. https://doi.org/10.1093/brain/awac366

Cole, R. D., Zimmerman, M., Matchanova, A., Kutlu, M. G., Gould, T. J., & Parikh, V. (2020). Cognitive rigidity and BDNF-mediated frontostriatal glutamate neuroadaptations during spontaneous nicotine withdrawal. *Neuropsychopharmacology*, *45*(5), 866–876. https://doi.org/10.1038/s41386-019-0574-6

Edemann-Callesen, H., Barak, S., Hadar, R., & Winter, C. (2020). Choosing the Optimal Brain Target for Neuromodulation Therapies as Alcohol Addiction Progresses—Insights From Pre-Clinical Studies. *Current Addiction Reports*, *7*(3), 237–244. https://doi.org/10.1007/s40429-020-00316-w

Eliason, M., Kalbande, P. P., & Saleem, G. T. (2024). Is non-invasive neuromodulation a viable technique to improve neuroplasticity in individuals with acquired brain injury? A review. *Frontiers in Human Neuroscience*, *18*. https://doi.org/10.3389/fnhum.2024.1341707

Evancho, A., Tyler, W. J., & McGregor, K. (2023). A review of combined neuromodulation and physical therapy interventions for enhanced neurorehabilitation. *Frontiers in Human Neuroscience*, *17*. https://doi.org/10.3389/fnhum.2023.1151218

Falade, J., Ajiboye, O., Olaogun, D., Anadu, V., Momodu, I., & Ola, O. (2025). Neuroanatomical Basis of Addiction: A narrative Review. *East African Journal of Neurological Sciences*, *4*(1), 63–79. https://doi.org/10.4314/eajns.v4i1.8

Ferreira, M., Matias, F., Abrunhosa, A., Martins, R., & Castelo-Branco, M. (2025). Evidence for Neuroplasticity in the Human Brain in Health and Disease: A Systematic Review Focusing on Molecular Imaging Using PET. *Neural Plasticity*, *2025*(1). https://doi.org/10.1155/np/9423232

Gazerani, P. (2025). The neuroplastic brain: current breakthroughs and emerging frontiers. *Brain Research*, *1858*. https://doi.org/10.1016/j.brainres.2025.149643

Gueguen, M. C., Schweitzer, E. M., & Konova, A. B. (2021). Computational theory-driven studies of reinforcement learning and decision-making in addiction: what have we learned? *Current Opinion in Behavioral Sciences*, *38*, 40–48. https://doi.org/10.1016/j.cobeha.2020.08.007

Guercio, L. A., Wimmer, M. E., Schmidt, H. D., Swinford-Jackson, S. E., Pierce, R. C., & Vassoler, F. M. (2020). Deep brain stimulation of the infralimbic cortex attenuates cocaine priming-induced reinstatement of drug seeking. *Brain Research*, *1746*. https://doi.org/10.1016/j.brainres.2020.147011

Guo, X., Yuan, Y., Su, X., Cao, Z., Chu, C., Lei, C., Wang, Y., Yang, L., Pan, Y., Sheng, H., Cui, D., Shao, D., Yang, H., Fu, Y., Wen, Y., Cai, Z., Lai, B., Chen, M., & Zheng, P. (2024). Different projection neurons of basolateral amygdala participate in the retrieval of morphine withdrawal memory with diverse molecular pathways. *Molecular Psychiatry*, *29*(3), 793–808. https://doi.org/10.1038/s41380-023-02371-x

Hammad, A. M., Alzaghari, L. F., Alfaraj, M., Lux, V., & Sunoqrot, S. (2024). Green Tea Polyphenol Nanoparticles Reduce Anxiety Caused by Tobacco Smoking Withdrawal in Rats by Suppressing Neuroinflammation †. *Toxics*, *12*(8). https://doi.org/10.3390/toxics12080598

Hassan, O., Phan, S., Wiecks, N., Joaquin, C., & Bondarenko, V. (2021). Outcomes of deep brain stimulation surgery for substance use disorder: a systematic review. *Neurosurgical Review*, *44*(4), 1967–1976. https://doi.org/10.1007/s10143-020-01415-y

He, L., Liao, Y., Wu, Q., & Liu, T. (2020). Association Between Brain-Derived Neurotrophic Factor Val66Met Polymorphism and Methamphetamine Use Disorder: A Meta-Analysis. *Frontiers in Psychiatry*, *11*. https://doi.org/10.3389/fpsyt.2020.585852

Iamjan, S. A., Thanoi, S., Watiktinkorn, P., Fachim, H., Dalton, C. F., Nudmamud-Thanoi, S., & Reynolds, G. P. (2021). Changes of exon IV DNA methylation are associated with methamphetamine dependence. *Epigenomics*, *13*(12), 953–965. https://doi.org/10.2217/epi-2020-0463

Jahn, F. S., Skovbye, M., Obenhausen, K., Jespersen, A. E., & Miskowiak, K. W. (2021). Cognitive training with fully immersive virtual reality in patients with neurological and psychiatric disorders: A systematic review of randomized controlled trials. *Psychiatry Research*, *300*. https://doi.org/10.1016/j.psychres.2021.113928

Jain, S. (2025). Neuroplasticity and Its Role in Occupational Rehabilitation. *2025 15th IEEE Integrated STEM Education Conference, ISEC 2025*. https://doi.org/10.1109/ISEC64801.2025.11147365

Kallupi, M., Kononoff, J., Melas, P. A., Qvist, J. S., de Guglielmo, G., Kandel, E. R., & George, O. (2022). Deep brain stimulation of the nucleus accumbens shell attenuates cocaine withdrawal but increases cocaine self-administration, cocaine-induced locomotor activity, and GluR1/GluA1 in the central nucleus of the amygdala in male cocaine-dependent rats. *Brain Stimulation*, *15*(1), 13–22. https://doi.org/10.1016/j.brs.2021.11.003

Keady, J. V., Hessing, M. C., Songrady, J. C., McLaurin, K., & Turner, J. R. (2024). Sex differences in contextual fear conditioning and extinction after acute and chronic nicotine treatment. *Biology of Sex Differences*, *15*(1). https://doi.org/10.1186/S13293-024-00656-6

Le, T. M., Potvin, S., Zhornitsky, S., & Li, C. S. R. (2021). Distinct patterns of prefrontal cortical disengagement during inhibitory control in addiction: A meta-analysis based on population characteristics. *Neuroscience and Biobehavioral Reviews*, *127*, 255–269. https://doi.org/10.1016/j.neubiorev.2021.04.028

Lenze, E. J., Nicol, G. E., Barbour, D. L., Kannampallil, T., Wong, A. W. K., Piccirillo, J., Drysdale, A. T., Sylvester, C. M., Haddad, R., Miller, J. P., Low, C. A., Lenze, S. N., Freedland, K. E., & Rodebaugh, T. L. (2021). Precision clinical trials: A framework for getting to precision medicine for neurobehavioural disorders. *Journal of Psychiatry and Neuroscience*, *46*(1), 97–110. https://doi.org/10.1503/jpn.200042

Liu, Q., & Yuan, T. (2021). Noninvasive brain stimulation of addiction: one target for all? *Psychoradiology*, *1*(4), 172–184. https://doi.org/10.1093/psyrad/kkab016

Lomas, C. (2024). Neurobiology, psychotherapeutic interventions, and emerging therapies in addiction: a systematic review. *Journal of Addictive Diseases*. https://doi.org/10.1080/10550887.2024.2440184

Lu, J., Tjia, M., Mullen, B., Cao, B., Lukasiewicz, K., Shah-Morales, S., Weiser, S., Cameron, L. P., Olson, D. E., Chen, L., & Zuo, Y. (2021). An analog of psychedelics restores functional neural circuits disrupted by unpredictable stress. *Molecular Psychiatry*, *26*(11), 6237–6252. https://doi.org/10.1038/s41380-021-01159-1

Mahoney, J. J., Haut, M. W., Hodder, S. L., Zheng, W., Lander, L. R., Berry, J. H., Farmer, D. L., Marton, J. L., Ranjan, M., Brandmeir, N. J., Finomore, V. S., Hensley, J. L., Aklin, W. M., Wang, G. J., Tomasi, D., Shokri-Kojori, E., & Rezai, A. R. (2021). Deep brain stimulation of the nucleus accumbens/ventral capsule for severe and intractable opioid and benzodiazepine use disorder. *Experimental and Clinical Psychopharmacology*, *29*(2), 210–215. https://doi.org/10.1037/pha0000453

Martínez-Fernández, D. E., Fernández-Quezada, D., Garzón-Partida, A. P., Aguilar-García, I. G., García-Estrada, J., & Luquin, S. (2024). The Effect of Eye Movement Desensitization and Reprocessing (EMDR) Therapy on Reducing Craving in Populations with Substance Use Disorder: A Meta-Analysis. *Brain Sciences*, *14*(11). https://doi.org/10.3390/brainsci14111110

Martinotti, G., Pettorruso, M., Montemitro, C., Ekhtiari, H., Hanlon, C. A., Spagnolo, P. A., Stein, E., & Di Giannantonio, M. (2020). Repetitive Transcranial Magnetic Stimulation in Addiction. In *Non Invasive Brain Stimulation in Psychiatry and Clinical Neurosciences* (pp. 135–160). Springer International Publishing. https://doi.org/10.1007/978-3-030-43356-7_11

McConnell, P. A., Garland, E. L., Zubieta, J. K., Newman-Norlund, R., Powers, S., & Froeliger, B. (2020). Impaired frontostriatal functional connectivity among chronic opioid using pain patients is associated with dysregulated affect. *Addiction Biology*, *25*(2). https://doi.org/10.1111/adb.12743

Mehta, D. D., Praecht, A., Ward, H. B., Sanches, M., Sorkhou, M., Tang, V. M., Steele, V. R., Hanlon, C. A., & George, T. P. (2024). A systematic review and meta-analysis of neuromodulation therapies for substance use disorders. *Neuropsychopharmacology*, *49*(4), 649–680. https://doi.org/10.1038/s41386-023-01776-0

Memos, N., Avila, J. A., Rodriguez, E., & Serrano, P. A. (2023). Synaptic remodeling of GluA1 and GluA2 expression in the nucleus accumbens promotes susceptibility to cognitive deficits concomitant with downstream GSK3β mediated neurotoxicity in female mice during abstinence from voluntary oral methamphetamine. *Addiction Neuroscience*, *8*. https://doi.org/10.1016/j.addicn.2023.100112

Moliner, R., Girych, M., Brunello, C. A., Kovaleva, V., Biojone, C., Enkavi, G., Antenucci, L., Kot, E. F., Goncharuk, S. A., Kaurinkoski, K., Kuutti, M., Fred, S. M., Elsilä, L. V., Sakson, S., Cannarozzo, C., Diniz, C. R. A. F., Seiffert, N., Rubiolo, A., Haapaniemi, H., … Castrén, E. (2023). Psychedelics promote plasticity by directly binding to BDNF receptor TrkB. *Nature Neuroscience*, *26*(6), 1032–1041. https://doi.org/10.1038/s41593-023-01316-5

Namba, M. D., Leyrer-Jackson, J. M., Nagy, E. K., Olive, M. F., & Neisewander, J. L. (2021). Neuroimmune Mechanisms as Novel Treatment Targets for Substance Use Disorders and Associated Comorbidities. *Frontiers in Neuroscience*, *15*. https://doi.org/10.3389/fnins.2021.650785

Ornell, F., Scherer, J. N., Schuch, J. B., Sordi, A. O., Halpern, S. C., Rebelatto, F. P., Bristot, G., Kapczinski, F., Roglio, V. S., Pechansky, F., Kessler, F. H. P., & von Diemen, L. (2023). Serum BDNF levels increase during early drug withdrawal in alcohol and crack cocaine addiction. *Alcohol*, *111*, 1–7. https://doi.org/10.1016/j.alcohol.2023.04.001

Peters, J., & Olson, D. E. (2021). Engineering Safer Psychedelics for Treating Addiction. *Neuroscience Insights*, *16*. https://doi.org/10.1177/26331055211033847

Quigley, J. A., Logsdon, M. K., Turner, C. A., Gonzalez, I. L., Leonardo, N. B., & Becker, J. B. (2021). Sex differences in vulnerability to addiction. *Neuropharmacology*, *187*. https://doi.org/10.1016/j.neuropharm.2021.108491

Raj, K., & Verdejo-Garcia, A. (2020). From impulses to compulsions. In *Cognition and Addiction: A Researcher’s Guide from Mechanisms Towards Interventions* (pp. 9–15). Elsevier. https://doi.org/10.1016/B978-0-12-815298-0.00002-2

Romanoff, S. R. (2022). Commentary on Mindfulness-Based Techniques for Behavioral Addiction. *Clinical Psychology: Science and Practice*, *29*(4), 397–399. https://doi.org/10.1037/cps0000109

Stankevich, Y. A., Popov, V. V., Bogomyakova, O. B., Vasilkiv, L. M., Tulupov, A. A., & Sagdeev, R. Z. (2024). VISUALIZATION OF BRAIN NEUROPLASTICITY IN THE ASPECT OF POST-STROKE REHABILITATION[ВИЗУАЛИЗАЦИЯ НЕЙРОПЛАСТИЧНОСТИ ГОЛОВНОГО МОЗГА В АСПЕКТЕ ПОСТИНСУЛЬТНОЙ РЕАБИЛИТАЦИИ]. *Complex Issues of Cardiovascular Diseases*, *13*(4), 214–228. https://doi.org/10.17802/2306-1278-2024-13-4-214-228

Takamatsu, Y., Inoue, T., Nishio, T., Soma, K., Kondo, Y., Mishima, T., Takamura, H., Okamura, M., & Maejima, H. (2024). Potential effect of physical exercise on the downregulation of BDNF mRNA expression in rat hippocampus following intracerebral hemorrhage. *Neuroscience Letters*, *824*. https://doi.org/10.1016/j.neulet.2024.137670

Tataranu, L. G., & Rizea, R. E. (2025). Neuroplasticity and Nervous System Recovery: Cellular Mechanisms, Therapeutic Advances, and Future Prospects. *Brain Sciences*, *15*(4). https://doi.org/10.3390/brainsci15040400

Tortora, C., Di Crosta, A., La Malva, P., Prete, G., Ceccato, I., Mammarella, N., Di Domenico, A., & Palumbo, R. (2024). Virtual reality and cognitive rehabilitation for older adults with mild cognitive impairment: A systematic review. *Ageing Research Reviews*, *93*. https://doi.org/10.1016/j.arr.2023.102146

Ulisse, K., Albitar, J., Aromin, J. T., & Berry, J. (2025). Emerging Interventions in Behavioral Addictions: A Narrative Review of Psychedelics and Neuromodulation. *Brain Sciences*, *15*(9). https://doi.org/10.3390/brainsci15090980

Wang, K., Li, Y., Zhang, T., Liu, H., & Luo, J. (2025). Potential benefits and mechanisms of physical exercise and rTMS in improving brain function in people with drug use disorders. *General Hospital Psychiatry*, *93*, 61–66. https://doi.org/10.1016/j.genhosppsych.2025.01.001

Wani, S. N., Grewal, A. K., Khan, H., & Singh, T. G. (2024). Elucidating the molecular symphony: unweaving the transcriptional & epigenetic pathways underlying neuroplasticity in opioid dependence and withdrawal. *Psychopharmacology*, *241*(10), 1955–1981. https://doi.org/10.1007/s00213-024-06684-9

Yang, A. Z., Tang, V. M., MacKillop, J., Boutet, A., Skelin, I., Vetkas, A., Fomenko, A., Sadeghi, A., Mehta, D., Sloan, M. E., Bhat, V., Milosevic, L., Kalia, S. K., Hamani, C., Lipsman, N., Valiante, T. A., Lozano, A. M., & Davidson, B. (2025). Shared Symptoms, Circuits, and Potential Solutions — Progress of Precision Lesioning for Obsessive-Compulsive Disorder as Rationale for Applications to Addiction. *Current Addiction Reports*, *12*(1). https://doi.org/10.1007/s40429-025-00684-1

Yin, R., Wang, S., & Wang, P. (2021). Effects of combined rTMS and exercise training on locomotor function and neuronal plasticity in rats with spinal cord injury[重复经颅磁刺激叠加运动训练对脊髓损伤大鼠运动功能和神经元可塑性的影响]. *Chinese Journal of Rehabilitation Medicine*, *36*(7). https://doi.org/10.3969/j.issn.1001-1242.2021.07.002

Zhang, L., Meng, S., Chen, W., Chen, Y., Huang, E., Zhang, G., Liang, Y., Ding, Z., Xue, Y., Chen, Y., Shi, J., & Shi, Y. (2021). High-Frequency Deep Brain Stimulation of the Substantia Nigra Pars Reticulata Facilitates Extinction and Prevents Reinstatement of Methamphetamine-Induced Conditioned Place Preference. *Frontiers in Pharmacology*, *12*. https://doi.org/10.3389/fphar.2021.705813

Zhang, R., Dang, W., Zhang, J., He, R., Li, G., Zhang, L., Wang, Z., Zong, H., Liu, N., & Jia, W. (2022). Methylation quantitative locus rs3758653 in the DRD4 gene is associated with duration from first heroin exposure to addiction. *Brain Research*, *1775*. https://doi.org/10.1016/j.brainres.2021.147746

Zhou, H., Hong, T., Chen, X., Su, C., Teng, B., Xi, W., Cadet, J. L., Yang, Y., Geng, F., & Hu, Y. (2024). Glutamate concentration of medial prefrontal cortex is inversely associated with addictive behaviors: a translational study. *Translational Psychiatry*, *14*(1). https://doi.org/10.1038/s41398-024-03145-x

Zhumakhanova, R., Zhaparkulova, N., Sharipova, S., Orazbayeva, N., Yeszhan, B., Zhaksybay, Z., Ossikbayeva, S., Akhmetova, A., & Ursheeva, B. (2024). Innovative achievements in the detection of synaptic plasticity and oxidative stress in mice: Precision imaging, improved biosensoring, and personalized interventions for neurological disorders. *Caspian Journal of Environmental Sciences*, *22*(3), 697–713. https://doi.org/10.22124/cjes.2024.7916

Zolali, E., McMahon, L. R., & Obeng, S. (2025). Effects of Alcohol and Opioids on Neuroregeneration. In *Tissue Repair and Regeneration: Elucidating Cellular and Molecular Mechanisms with Therapeutic Implications* (pp. 263–280). Springer Nature Switzerland. https://doi.org/10.1007/978-3-031-93677-7_12
